# The mechanism of fashion consciousness influencing Gen Z’s Willingness to continue using slow fashion apparel in a sustainable context

**DOI:** 10.1371/journal.pone.0357599

**Published:** 2026-09-03

**Authors:** Jiayu Zhu, Anding Liu

**Affiliations:** 1 Wuhan Textile University, School of Fashion, Wuhan, China; 2 Academy for Forging a Strong Sense of The Zhonghuaminzu Community and Advancing the Sinicizationof Religion in China, Wuhan, China; University of Mpumalanga, SOUTH AFRICA

## Abstract

To explore the mechanisms underlying Gen Z consumers’ willingness to continue using slow fashion apparel, this study constructs a conceptual model based on SOR theory. The model considers personal and social fashion consciousness as stimulus variables, aesthetic, emotional, social, and green values as organism variables, and willingness to continue using as the response variable. Data were collected through a questionnaire based on a 7-point Likert scale, yielding 311 valid responses. SPSS and JASP were used to assess reliability and validity and examine direct and mediation effects. The results indicate that personal fashion consciousness, social value, and green value are all significantly and positively associated with willingness to continue using slow fashion apparel. Personal fashion consciousness is positively associated with perceived aesthetic and emotional values, whereas social fashion consciousness is positively associated with perceived social and green values. Furthermore, social and green values mediate the relationship between social fashion consciousness and willingness to continue using slow fashion apparel. However, the mediating effects of aesthetic and emotional values are not significant. This study suggests that in the context of sustainable consumption, value-related factors may better explain long-term use behavior than aesthetic experiences alone. These findings suggest that slow fashion brands should support individual expression and sustainability-related meanings and enhance Gen Z consumers’ perceptions of green and social value, thereby encouraging continued use of slow fashion apparel and promoting sustainable fashion development.

## Introduction

Amid increasing ecological pressure and the continued depletion of natural resources, sustainable consumption has increasingly become key pathway toward social development. Since the beginning of the 21st century, fast fashion has fundamentally transformed the fashion industry while also generating negative environmental and social impacts throughout the entire product life cycle [1]. The rapid turnover of clothing styles has causes large quantities of still-usable garments to be discarded as outdated, resulting in resource waste, environmental pollution, and emissions from incineration. As an alternative to fast fashion, the concept of slow fashion was introduced by British designer Kate Fletcher in 2007 through an article published in *The Ecologist*. It seeks to reconcile the tension between fashion and sustainability across various stages, including material sourcing, design, production, distribution, and consumption [2]. Slow fashion apparel emphasizes quality, durability, and timeless design. It advocates extending garment lifespans, which is consistent with current green consumption trends.

Generation Z (Gen Z) generally refers to “digital natives” born after widespread public access to the internet [3]. Because no consensus has been reached regarding its birth-year boundaries, this study defines Gen Z as individuals born between 1995 and 2009, based on the Chinese sociocultural context and relevant domestic research [4]. As an important force in fashion consumption, Gen Z values individual expression and personal style, demonstrating strong personal fashion consciousness. At the same time, they show considerable concern for climate change and social issues, reflecting pronounced social fashion consciousness and environmental responsibility [5]. However, under the influence of the fast-paced visual culture of social media, a gap remains between their sustainable consumption attitudes and actual behavior, making it difficult for them to continue using slow fashion apparel over the long term [6]. Many consumers purchase slow fashion products with pro-environmental intentions, yet frequently leave garments unused or discard them prematurely, often while they are still physically functional—due to rapidly shifting trends or aesthetic fatigue. Therefore, examining the association between fashion consciousness on Gen Z consumers’ willingness to continue using slow fashion apparel can deepen understanding of their slow fashion consumption behavior.

Extensive research has explored sustainability within the apparel industry from diverse perspectives. From the perspective of consumer behavior, studies have focused on purchase intention toward sustainable apparel [7], willingness to pay a premium [8], and green apparel consumption behavior [9]. From the perspective of production and supply chains, emphasis has been placed on circular supply chains and corporate social responsibility [10,11]. From the perspective of design and aesthetics, studies have highlighted sustainable design approaches such as “one garment, multiple outfits” and zero-waste design [12]. However, current research primarily centers on consumers’ initial purchase intentions or behaviors, with insufficient attention given to the factors associated with post-purchase willingness to continue using and its underlying mechanisms. The environmental benefit of slow fashion lies fundamentally in extending garment lifecycles. Without sustained use, purchasing becomes merely another form of resource stockpiling. Furthermore, existing research has examined the relationship between fashion consciousness on consumer behavior but often treats fashion consciousness as a singular construct, with limited discussion of its multiple dimensions. For Gen Z, who simultaneously emphasize individual expression and social responsibility, the roles of personal and social fashion consciousness in slow fashion consumption may diverge. Accordingly, these distinct mechanisms require further examination and empirical validation. This study, therefore, employs the SOR framework to construct a model: “Fashion Consciousness → Perceived Value → Willingness to Continue Using.” This research aims to examine the associations between different dimensions of fashion consciousness on Gen Z consumers’ willingness to continue using slow fashion apparel and to test the mediating role of perceived value. Applying the SOR model to this field offers significant implications for prolonging garment lifecycles, informing targeted product planning by slow fashion brands, and advancing the industry’s green transformation.

### Theoretical models and research hypotheses

#### SOR theoretical model.

The SOR (Stimulus–Organism–Response) theory, first proposed by Mehrabian in 1974, emphasizes that external environmental shape influence an organism’s behavioral and psychological states, which are then manifested through its responses [13]. Compared with the traditional “S-R” model, the SOR theory introduces the “Organism” variable to represent internal psychological processes. It stresses that individual behavioral responses are not directly determined by stimuli but instead emerge through the processing of internal states, including cognition, emotion, attitudes, and value evaluations.

In consumer behavior research, the SOR theory has been widely used to explain how consumers move from external stimuli to behavioral responses. In the context of fashion and sustainable consumption, Eroglu et al. [14] found that fashion consciousness is closely associated with consumers’ pleasure, arousal, attitudes, satisfaction, and purchasing behavior. Ren et al. [15] showed that fashion consciousness has a positive association with consumers’ online purchase intention for sustainable apparel. This suggests that fashion consciousness is an important antecedent of sustainable apparel consumption behavior. In this study, fashion consciousness is regarded as a stimulus factor. It reflects an individual’s sensitivity to fashion information and identification with social fashion norms. Perceived value is treated as the organism variable and reflects consumers’ overall evaluation of product benefits, costs, and meanings after exposure to stimuli [16]. It is therefore well suited to represent the organism component of the SOR model. Previous studies have also shown that perceived value can mediate the relationship between experience quality and behavioral intention. It can also connect stimulus factors with purchase intention or willingness to continue using within the SOR framework [17]. Willingness to continue using is treated as the response variable, reflecting consumers’ tendency to use slow fashion apparel over the long term after purchase and serving as an important indicator of sustainable consumption behavior. Therefore, positioning perceived value between fashion consciousness and willingness to continue using not only follows the basic logic of the SOR theory, but also helps clarify the relationships among fashion consciousness, perceived value, and willingness to continue using slow fashion apparel. Based on this framework, this study proposes the research hypotheses and conducts empirical analysis.

### The Relationship Between Fashion Consciousness and Willingness to Continue Using

Fashion consciousness refers to consumers’ perceptions and attitudes toward fashion. It reflects consumers’ lifestyles and values [18]. Fashion consciousness manifests as both individual aesthetic preferences and self-expression needs, as well as a focus on social fashion norms and others’ evaluations. Consequently, relevant studies categorize fashion consciousness into two dimensions: personal fashion consciousness and social fashion consciousness [19]. Personal fashion focuses on individual aesthetic preferences, self-fulfillment, and personal expression. Social fashion consciousness, conversely, emphasizes attention to fashion trends, group norms, and others’ evaluations. Consumers with higher personal fashion consciousness tend to perceive slow fashion apparel as a long-term choice aligned with their lifestyle, aesthetic orientation, and values, thereby being more likely to demonstrate a relatively stable willingness to continue using it [20]. In the context of slow fashion and sustainable apparel consumption, consumers with stronger social fashion consciousness tend to attach great importance to external evaluations and social norms in career development and social interactions. They seek to demonstrate their values stance and sense of social responsibility to others through such apparel consumption [21]. Therefore, social fashion consciousness may directly be positively associated with consumers’ willingness to continue using slow fashion apparel even without relying on specific value judgments. Based on this, the study further proposes the following hypotheses:

H1: Personal fashion consciousness is significantly and positively associated with the willingness to continue using slow fashion apparel.

H2: Social fashion consciousness is significantly and positively associated with the willingness to continue using slow fashion apparel.

### The relationship between perceived value and willingness to continue using

The categorization of dimensions and construction of perceptual models are central to research on methods for evaluating user perceptions [22]. By examining the relationships between different value dimensions on behavioral intention, researchers can more clearly identify the key sources of consumers’ value evaluation and provide a basis for firms to optimize the value offered by their products. Consumer perceived value refers to an individual’s overall evaluation of a product’s benefits and costs, encompassing functional, service, economic, and social aspects [23]. Sheth et al. [24] classified perceived value into functional, social, emotional, cognitive, and conditional value. Flint et al. [25] argued that beyond simple trade-offs between perceived quality and price, perceived value involves complex trade-offs among benefits and costs, including time, risk, and effort. Wang et al. [26] categorized customer perceived value dimensions into functional, emotional, social, cognitive, conditional, and cost-related value. According to perceived value theory, consumers form an overall perception of a product’s utility through multidimensional value judgments during use and evaluation. Such value perceptions are closely related to subsequent usage behavior. Drawing on previous studies and the characteristics of slow fashion apparel, this study argues that consumers’ perceived aesthetic, emotional, social, and green values are important factors associated with their willingness to continue using such products.

Aesthetic value refers to consumers’ subjective evaluation of the visual design, style expression, and overall aesthetic appeal of slow fashion apparel. Ju-Hyun Ro et al. [27] noted that slow fashion embodies cyclical, sustainable, moderate, and expressive forms of beauty. These aesthetic attributes may contribute to consumers’ affection for such apparel. In addition, studies by Jung [28] and Şener [29] on slow fashion attributes, perceived value, and behavioral intention suggest that higher perceived aesthetic value is associated with higher consumers’ overall evaluations of slow fashion apparel and their satisfaction with its use, thereby being associated with greater their willingness to continue using such apparel in the future. Therefore, this study proposes the following hypothesis:

H3: Aesthetic value is significantly and positively associated with the willingness to continue using slow fashion apparel.

Emotional value refers to the pleasure, satisfaction, self-identification, sense of self-identity, and emotional connection that consumers obtain when using slow fashion apparel. Sweeney et al. [30] identified emotional value as an important dimension for explaining consumer attitudes and behavioral intentions. Wu [31] examined the relationships between different dimensions of customer perceived value and consumers’ intention to purchase green apparel, finding that emotional value and green value were most strongly associated with purchase intention. Liao [32] further found that emotional, social, price, and quality values were positively associated with attitudes toward slow fashion. Among these dimensions, emotional value had the strongest association, and attitudes toward slow fashion further had a positive association with purchase intention. Therefore, the stronger the positive emotional experiences consumers derive from using slow fashion apparel, the greater their willingness to continue using it may be. Based on this reasoning, this study proposes the following hypothesis:

H4: Emotional value is significantly and positively associated with the willingness to continue using slow fashion apparel.

Social value refers to the extent to which consumers obtain social recognition, express their identities, and communicate their values through slow fashion apparel. Consumers can communicate their personal aesthetics, lifestyle, and values to others through apparel style, brand philosophy, and consumption choices. Previous slow fashion research has shown that social value is positively associated with attitudes toward slow fashion, and that subjective norms are positively associated with slow fashion purchase intention. This indicates that others’ evaluations and social recognition are relevant to slow fashion consumption [32]. Liang et al. [33] also found, based on green apparel information, that social value is significantly and positively associated with willingness to engage in sustainable consumption behavior. Therefore, when slow fashion apparel provides opportunities for consumers to obtain social recognition and express their social identity, their willingness to continue using it may increase. Based on this reasoning, this study proposes the following hypothesis:

H5: Social value is significantly and positively associated with the willingness to continue using slow fashion apparel.

Green value refers to consumers’ value judgment of slow fashion apparel in terms of environmental protection, waste reduction, and sustainable development. Arora et al. [34] found that green value can positively be positively associated with purchase intention through consumers’ positive attitudes toward sustainable apparel. Li et al. [35] also noted that, compared with conventional apparel, consumers showed higher perceived value, purchase intention, and repurchase intention toward sustainable apparel. Thus, when consumers perceive a higher level of green value in slow fashion apparel, they are not only more likely to demonstrate purchase intention, but also more likely to maintain their willingness to continue using it during subsequent use. Based on this reasoning, this study proposes the following hypothesis:

H6: Green value is significantly and positively associated with the willingness to continue using slow fashion apparel.

### The relationship between fashion consciousness and perceived value

Consumer perceived value is the overall assessment of the net benefits of a product or service based on consumers’ own judgment and is an important is associated with behavioral intention [36]. Existing studies indicate that consumers’ fashion consciousness not on is associated with their evaluation of apparel’s external attributes, but also relates to their overall perception of a product’s intrinsic value and symbolic meaning [37]. Therefore, fashion consciousness can be regarded as an important factor associated with perceived value in the context of slow fashion apparel. Based on the definitions of the constructs and their conceptual correspondence with the value dimensions, this study focuses on theoretically more direct and proximal pathways.

Consumers with higher personal fashion consciousness typically focus more on the uniqueness of apparel in terms of silhouette, color, and design language, and tend to view apparel as a key medium for expressing individuality and aesthetic taste. O’Cass [38] found that the congruence between consumers’ self-image and product image is positively associated with their involvement in fashion apparel. This suggests that consumers use apparel to construct and express their self-image. Hourigan et al. [39] also noted that fashion apparel involvement reflects the importance and meaning of apparel in consumers’ lives. For slow fashion apparel, features such as classic design, material quality, authenticity, and uniqueness are more likely to be recognized and evaluated by consumers with higher personal fashion consciousness. At the same time, apparel also has functions related to self-expression, emotional experience, and identity construction. When consumers perceive that an apparel style is consistent with their aesthetic preferences, personality traits, and ideal image, they are more likely to experience pleasure, satisfaction, and emotional connection during wearing and use. Domingos et al. [40], in their review of slow fashion consumer behavior, pointed out that slow fashion consumption is closely related to consumers’ values, attitudes, and motivations. Liu et al. [41] further found a positive link between slow fashion and consumer well-being. This indicates that slow fashion apparel not only has functional and environmental attributes, but may also provide consumers with positive emotional experiences. Therefore, the stronger consumers’ personal fashion consciousness is, the more likely they are to perceive higher aesthetic value from the design concept of slow fashion apparel. They are also more likely to obtain self-expression, aesthetic pleasure, and emotional satisfaction during use, thereby developing a higher perception of emotional value. Although consumers with stronger personal fashion consciousness may also gain social recognition through personalized apparel choices, research on consumer identity suggests that individual preferences must be conveyed through socially visible product choices, self-presentation, and identity signals before they can be translated into evaluations by others and social identity benefits [42]. Therefore, the relationship between personal fashion consciousness and social value may depend on additional mechanisms, such as identity presentation, social visibility, and evaluations by others, rather than arising directly from personal fashion consciousness alone. Based on this reasoning, the following hypotheses are proposed:

H7: Personal fashion consciousness is significantly and positively associated with the aesthetic value of slow fashion apparel.

H8: Personal fashion consciousness is significantly and positively associated with the emotional value of slow fashion apparel.

Consumers with higher social fashion consciousness are more likely to understand apparel consumption from the perspectives of others’ recognition, social norms, and value expression. Castagna et al. [43] found in their study on self-signaling in slow fashion that when slow fashion consumption conveys signals of nonconformity, pro-environmental concern, and frugality, consumers tend to engage in more positive word-of-mouth and develop stronger perceptions of status. Therefore, the stronger consumers’ social fashion consciousness is, the more likely they are to view slow fashion apparel as a way to gain social recognition, build a positive social image. This may be associated with a higher perception of social value. In addition, slow fashion apparel emphasizes waste reduction, product life extension, and environmental responsibility. These characteristics are closely aligned with sustainable and green apparel in terms of environmental friendliness and responsible consumption. Kang et al. [44], in their study of environmentally sustainable textile and apparel consumption, found that consumer knowledge, perceived consumer effectiveness, and perceived personal relevance are positively associated with consumers’ attitudes and behavioral intentions toward sustainable apparel. Thus, consumers with higher social fashion consciousness are more likely to recognize the environmental significance of social responsibility and sustainable consumption norms. This may further contribute to their perceptions of social value and green value.

However, consumers with stronger social fashion consciousness may also experience positive emotions, such as pride and satisfaction, when their consumption choices conform to social norms, gain recognition from others, or demonstrate environmental responsibility. Such emotional benefits are usually related to consumers’ perceptions of the moral significance of their behavior, fulfillment of responsibility, or social recognition. Venhoeven et al. [45] noted that sustainable behavior can be associated with positive emotions because consumers regard it as morally meaningful. Research on the warm-glow effect has also shown that engaging in green or prosocial behavior can be related to intrinsic emotional rewards [46]. Therefore, the relationship between social fashion consciousness and emotional value may arise through psychological mechanisms such as social recognition, perceived green value, or moral satisfaction, rather than through a proximal mechanism by which social fashion consciousness directly affects emotional value. Based on this reasoning, the following hypotheses are proposed:

H9: Social fashion consciousness is significantly and positively associated with the social value of slow fashion apparel.

H10: Social fashion consciousness is significantly and positively associated with the green value of slow fashion apparel.

### The mediating role of perceived value

Based on the SOR theory, individuals first form internal psychological perceptions and evaluations in response to external stimuli, which subsequently are reflected in their behavioral responses. Wang et al. [47] found that perceived value has a significant mediating effect between consumer experience quality and behavioral intention. This indicates that consumers’ value evaluation of a product or service is an important psychological mechanism through which experience is transformed into behavior. Yu et al. [48] showed that consumers’ perceived value not only positively is positively associated with their product evaluation and purchase intentions, but is also closely related to subsequent green consumption behavior. Chen et al. [49], in the context of heritage tourism, found that experience quality can further be associated with tourists’ behavioral intentions through perceived value and satisfaction. This suggests that perceived value is an important mediating variable.

Based on the previous analysis, personal fashion consciousness may be associated with consumers’ multidimensional value judgments regarding slow fashion apparel. The perceived aesthetic, emotional, social, and green values formed by consumers further are associated with their willingness to continue using such apparel. Therefore, fashion consciousness may not only directly be associated with willingness to continue using but also exert an indirect association through perceived value. Specifically, personal fashion consciousness focuses on aesthetic experience, self-expression, and emotional satisfaction. Therefore, its effect on consumers’ willingness to continue using slow fashion apparel may mainly operate through their perceptions of aesthetic value and emotional value. In contrast, social fashion consciousness emphasizes consumers’ sensitivity to social norms, group identification, and others’ evaluations. Its effect on willingness to continue using is more likely to be transmitted through their perceptions of social value and green value. Based on this reasoning, the following hypotheses are proposed:

H11: Aesthetic value mediates the relationship between personal fashion consciousness and willingness to continue using slow fashion apparel.

H12: Emotional value mediates the relationship between personal fashion consciousness and willingness to continue using slow fashion apparel.

H13: Social value mediates the relationship between social fashion consciousness and willingness to continue using slow fashion apparel.

H14: Green value mediates the relationship between social fashion consciousness and willingness to continue using slow fashion apparel.

### Theoretical model

**Fig 1:**
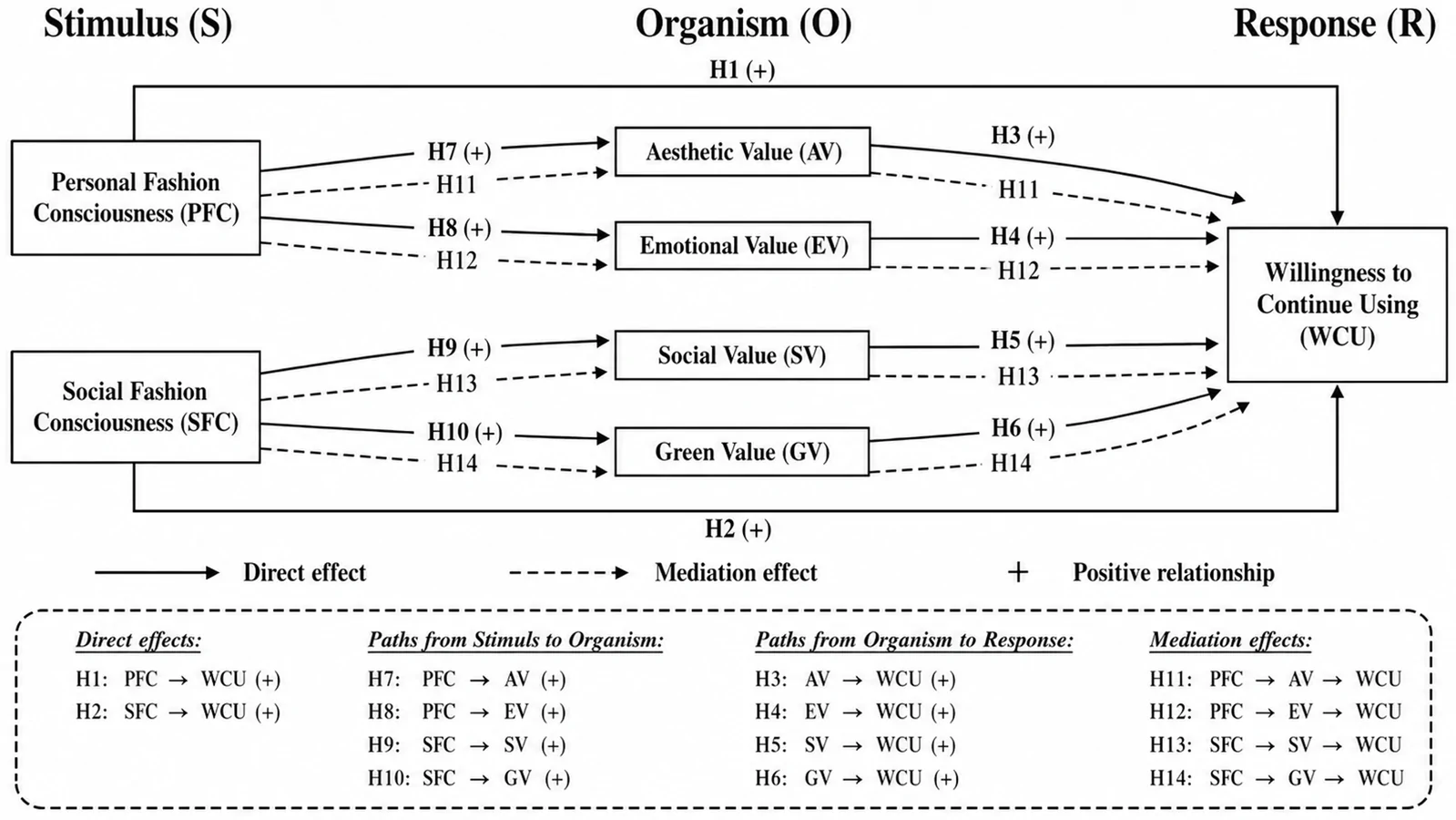
Research Model. The proposed SOR model illustrates the hypothesized relationships among fashion consciousness, perceived value, and continuous usage intention. H1–H14 indicate the direct and mediation hypotheses.

Based on the SOR framework, this study constructs a conceptual model examining the relationship between fashion consciousness and willingness to continue using slow fashion apparel, with perceived value as the mediating variable, as shown in Fig 1.

### Research design

#### Questionnaire design and variable measurement.

The questionnaire consists of three sections. The first section includes screening questions. These questions were used to identify eligible participants based on their birth year and their awareness of or purchase experience with slow fashion. The second section contains the measurement scales for the study variables. All items were measured using a 7-point Likert scale, ranging from 1 (“strongly disagree”) to 7 (“strongly agree”). Compared with a 5-point scale, a 7-point scale provides greater discriminatory power. It helps capture more subtle differences in respondents’ fashion consciousness, perceived value, and willingness to continue using. At the same time, it does not substantially increase the response burden. Therefore, it was considered appropriate for the questionnaire used in this study. The third section collects demographic information. The measurement items for all variables were adapted from established. The items were appropriately adapted to the slow fashion apparel context to improve the clarity and contextual applicability of the questionnaire. The measurement items and their references are shown in Table 1.

**Table 1:** Scale items and references.

Variable	Dimension	Code	Measurement Item	Reference Source
Fashion Consciousness	Personal Fashion Consciousness	PFC1	I place great emphasis on expressing my unique personal style in my everyday attire.	Ren et al. [15]
		PFC2	Rather than blindly following trends, I prioritize whether clothing aligns with my personal style.	
		PFC3	I usually express my aesthetic taste and personality through my clothing choices.	
		PFC4	I am confident in my aesthetic judgment when selecting clothing.	
	Social Fashion Consciousness	SFC1	I am highly attentive to the opinions of my social circles regarding my clothing choices.	Rieke et al. [50]
		SFC2	I often refer to outfit inspiration on social media to guide my purchasing decisions.	
		SFC3	I consider the aesthetic preferences of groups I identify with when choosing clothing.	
		SFC4	I hope to make a good first impression on others by wearing stylish clothing.	
Perceived Value	Aesthetic Value	AV1	I perceive the design style of slow fashion apparel to be classic and aesthetically pleasing.	Chi and Chen [51]
		AV2	The tailoring, color combinations, or patterns of slow fashion clothing provide me with aesthetic enjoyment.	
		AV3	Compared with fast fashion, I find slow fashion clothing possesses a timeless beauty.	
		AV4	I believe the style of slow fashion clothing possesses enduring appeal.	
	Emotional Value	EV1	Wearing slow fashion clothing usually makes me feel happy and relaxed.	Li and Xu [36]
		EV2	I tend to develop an emotional attachment to the slow fashion clothing I purchase, making it psychologically difficult for me to part with these garments.	
		EV3	The brand stories or designer philosophies behind slow fashion clothing often resonate with me emotionally.	
		EV4	I believe slow fashion apparel provides enduring emotional value rather than merely short-term utility.	
	Social Value	SV1	Wearing slow fashion clothing helps enhance my image in social settings.	Liang and Xu [33]
		SV2	Such clothing allows me to appear “tasteful” or “discerning” among friends or peers.	
		SV3	Choosing slow fashion clothing helps me create a positive impression in the eyes of others.	
		SV4	This type of clothing helps me better integrate into the social circles I aspire to be a part of.	
	Green Value	GV1	I highly value the resource-saving and sustainable attributes inherent in slow fashion apparel.	Aprianingsih et al. [52]
		GV2	The concept of slow fashion aligns with my values regarding environmental protection and social responsibility.	
		GV3	Buying slow fashion clothing makes me feel like I’m doing my part to protect the environment.	
		GV4	Choosing slow fashion clothing helps strengthen my own environmental awareness and may influence others’ views on environmental protection.	
Continued use	Willingness to Continue Using	WCU1	I am willing to wear my current slow fashion apparel consistently over a long period.	van Gogh et al. [53]
		WCU2	Even when new styles emerge, I tend to keep and continue wearing my old slow fashion garments.	
		WCU3	I am willing to invest the effort to maintain or repair slow fashion garments to extend their lifespan.	
		WCU4	I tend to incorporate slow fashion pieces as staples in my everyday wardrobe and wear them frequently.	

### Sample selection and data collection

The target population of this study was consumers born between 1995 and 2009 who had experience purchasing or using slow fashion apparel. Because accurate data on the size of this population were unavailable, it was treated as a l large population when estimating the required sample size. An a priori statistical power analysis was conducted using G*Power 3.1. Based on the research model, willingness to continue using (WCU) was considered the primary endogenous variable, with six predictors (PFC, SFC, AV, EV, SV, and GV). Therefore, the number of predictors was set to six. Following the recommendations of Faul et al. [54], the significance level was set at α = 0.05, statistical power at 1 − β = 0.80, and the expected effect size at f^2^ = 0.05. The analysis indicated a minimum required sample size of 279.

This study utilized the online survey platform “WENJUANXING” to distribute and collect questionnaires. The survey was conducted from January 5 to February 10, 2026. A total of 340 questionnaires were collected. Responses were excluded if respondents were outside the 1995–2009 birth-year range, lacked relevant experience, completed the questionnaire in less than 100 seconds, failed the attention checks, or exhibited patterned responding. After data screening, 311 valid questionnaires remained. This number exceeded the minimum sample size requirement, and the valid response rate was 91.47%. Therefore, the sample size was sufficient for the subsequent empirical analysis. All participants were informed of the research purpose, the voluntary nature of participation, and the confidentiality of their responses. Informed consent was obtained before the survey. A consent statement was presented on the first page of the questionnaire, and participants indicated their implied consent by clicking the “Start” button to continue. No personally identifiable information was collected. All data were analyzed anonymously to protect respondents’ privacy.

### Empirical analysis and hypothesis testing

#### Descriptive statistical analysis.

The descriptive statistics are presented in Table 2. Regarding gender distribution, females account for 55.0% and males for 45.0%, indicating a relatively balanced distribution with a slightly higher proportion of female respondents. This aligns with the higher female involvement typically observed in apparel consumption research. Regarding respondents’ status, students account for 26.0%, entry-level professionals for 32.5%, experienced professionals for 35.4%, and freelancers/others for 6.1%. Overall, the sample mainly comprised young people with diverse educational and occupational backgrounds. The distribution of monthly disposable income was relatively diverse: 15.4% reported less than ¥1,500, 29.3% reported ¥1,501–3,000, 16.1% reported ¥3,001–5,000, 22.2% reported ¥5,001–8,000, and 17.0% reported more than ¥8,000. Regarding slow fashion apparel purchase frequency, occasional buyers accounted for 50.5%, sometimes buyers accounted for 37.9%, and frequent buyers accounted for 11.6%. This indicates that respondents had some experience with slow fashion consumption, although the majority were low-to-medium frequency participants. Overall, the sample characteristics align with expectations, and the data were suitable for subsequent analysis.

**Table 2:** Descriptive statistical analysis of the sample.

Indicator Category	Characteristics	Frequency (n)	Percentage (%)
Gender	Male	140	45.0%
	Female	171	55.0%
Status	Students	81	26.0%
	Entry-level Professionals (1–3 years of work)	101	32.5%
	Experienced Professionals (Over 3 years of work)	110	35.4%
	Freelancers/ Others	19	6.1%
Monthly Disposable Income	Under ¥1,500	48	15.4%
	¥1,501–3,000	91	29.3%
	¥3,001–5,000	50	16.1%
	¥5,001–8,000	69	22.2%
	Over ¥8,000	53	17.0%
Slow Fashion Purchase Frequency	Occasional (1–2 times)	157	50.5%
	Sometimes (3–5 times)	118	37.9%
	Frequent (6 times or more)	36	11.6%

### Common method bias and multicollinearity tests

To assess potential common method variance, Harman’s single-factor test was conducted. The results showed that seven factors with eigenvalues greater than 1 were extracted. Before rotation, the first factor accounted for 35.633% of the total variance, which was below the threshold of 40%, indicating that this study did not suffer from serious common method bias. To further examine whether multicollinearity existed in the model, tolerance values and variance inflation factors (VIFs) were calculated. The tolerance values of all variables ranged from 0.539 to 0.667, all exceeding 0.10, while the VIF values ranged from 1.500 to 1.856, all below 3. These results indicate that there was no serious multicollinearity among the predictor variables, allowing the subsequent analyses to be conducted.

### Reliability and validity testing

To test the reliability and validity of the scale, this study used Cronbach’s α coefficient, exploratory factor analysis, and confirmatory factor analysis. Cronbach’s α coefficient was used to examine the internal consistency of the scale. Exploratory factor analysis was used to determine the correspondence between the items and their latent variables. Confirmatory factor analysis was further used to examine the construct validity, convergent validity, and discriminant validity of the measurement model.

In general, Cronbach’s α values and composite reliability values should both be greater than 0.700, which indicates that the scale has good internal consistency and stability [55]. The results from SPSS 27 indicated that the overall Cronbach’s α of the scale was 0.932. As shown in Table 3, the Cronbach’s α coefficients of all variables were higher than 0.800. Therefore, the scale demonstrated good reliability.

**Table 3:** Reliability, factor loadings, and convergent validity of the measurement scales.

Variable	Item	EFA Factor Loading	Standardized Factor Loading	Cronbach’s α	CR	AVE
Personal Fashion Consciousness(PFC)	PFC1	0.789	0.803	0.862	0.841	0.617
	PFC2	0.725	0.775			
	PFC3	0.772	0.752			
	PFC4	0.761	0.812			
Social Fashion Consciousness(SFC)	SFC1	0.787	0.781	0.861	0.837	0.617
	SFC2	0.746	0.794			
	SFC3	0.780	0.729			
	SFC4	0.742	0.833			
Aesthetic Value(AV)	AV1	0.719	0.745	0.854	0.828	0.605
	AV2	0.732	0.777			
	AV3	0.769	0.786			
	AV4	0.729	0.801			
Emotional Value(EV)	EV1	0.781	0.795	0.867	0.826	0.598
	EV2	0.717	0.799			
	EV3	0.761	0.738			
	EV4	0.751	0.761			
Social Value(SV)	SV1	0.745	0.835	0.839	0.823	0.587
	SV2	0.766	0.702			
	SV3	0.794	0.766			
	SV4	0.739	0.754			
Green Value(GV)	GV1	0.786	0.784	0.865	0.832	0.604
	GV2	0.756	0.772			
	GV3	0.775	0.793			
	GV4	0.804	0.760			
Willingness to Continue Using(WCU)	WCU1	0.803	0.781	0.861	0.829	0.606
	WCU2	0.785	0.799			
	WCU3	0.790	0.766			
	WCU4	0.793	0.766			

**Note:** The EFA factor loadings represent the primary loadings of each item on its corresponding dimension after rotation. The extraction method was principal component analysis, and the rotation method was Kaiser normalized varimax rotation. The standardized factor loadings, CR, and AVE were obtained from the confirmatory factor analysis results. CR refers to composite reliability, and AVE refers to average variance extracted.

Before conducting exploratory factor analysis, the KMO test and Bartlett’s test of sphericity were performed on the sample data. In general, a KMO value greater than 0.600 and a p-value less than 0.050 in Bartlett’s test indicate that the data are suitable for factor analysis. As shown in Table 4, the questionnaire data met these criteria.

**Table 4:** KMO and Bartlett’s Test.

Test Category		Value
KMO Measure of Sampling Adequacy	0.922	
Bartlett’s Test of Sphericity	Approx. Chi-Square	4726.355
	df	378
	Sig. (p-value)	0.000

Then, principal component analysis was used to extract the factors, and Kaiser normalized varimax rotation was applied. The results showed that seven factors had initial eigenvalues greater than 1, which was consistent with the number of variable dimensions in the questionnaire. The cumulative variance explained was 71.026%, exceeding the 60% threshold. As shown in Table 3, the primary loading of each item on its corresponding dimension was greater than 0.700. This suggests that the items were well associated with their corresponding latent variables. Therefore, the exploratory factor analysis results were satisfactory.

Confirmatory factor analysis mainly included assessments of construct validity, convergent validity, and discriminant validity. JASP software was used for the analysis. The construct validity of the measurement model was evaluated using model fit indices. As shown in Table 5, all model fit indices met the recommended standards, indicating that the overall measurement model had a good fit.

**Table 5:** Goodness-of-fit indices for the measurement model.

Fit Index	Measurement Model Fit	Recommended Criteria
$\chi^{\text{2}}/df$	1.177	1 ~ 3
RMSEA	0.024	< 0.08
SRMR	0.034	< 0.08
TLI	0.993	> 0.90
CFI	0.994	> 0.90

Next, the convergent validity of the scale was tested using standardized factor loadings, composite reliability (CR), and average variance extracted (AVE). In confirmatory factor analysis, standardized factor loadings greater than 0.500, AVE values greater than 0.500, and CR values greater than 0.700 generally indicate good convergent validity. As shown in Table 3, all indicators met the recommended thresholds, indicating that the scale had good convergent validity.

Finally, discriminant validity was assessed using the Heterotrait–Monotrait Ratio (HTMT). As shown in Table 6, the HTMT values between the latent variables ranged from 0.356 to 0.610, all below the recommended threshold of 0.85. This indicates that the latent variables had good discriminant validity. Overall, the scale used in this study showed good reliability, construct validity, convergent validity, and discriminant validity, and therefore met the requirements for subsequent empirical analysis.

**Table 6:** HTMT discriminant validity.

Constructs	PFC	SFC	AV	EV	SV	GV	WCU
PFC	—						
SFC	0.602	—					
AV	0.597	0.610	—				
EV	0.473	0.605	0.597	—			
SV	0.424	0.371	0.586	0.505	—		
GV	0.449	0.356	0.430	0.571	0.520	—	
WCU	0.486	0.379	0.408	0.413	0.436	0.469	—

### Correlation analysis

To further examine the relationships among the variables, correlation analysis was conducted, and the results are presented in Table 7. All study variables were significantly and positively correlated with one another (p < 0.01). Specifically, personal fashion consciousness and social fashion consciousness were significantly and positively correlated with willingness to continue using. Aesthetic value, emotional value, social value, and green value were also significantly and positively correlated with willingness to continue using. These results were generally consistent with the expected directions of the research hypotheses and provided preliminary evidence for further structural equation modeling and mediation analyses.

**Table 7:** Correlation analysis results.

Variable	PFC	SFC	AV	EV	SV	GV	WCU
PFC	1						
SFC	0.517**	1					
AV	0.513**	0.522**	1				
EV	0.409**	0.522**	0.513**	1			
SV	0.361**	0.316**	0.498**	0.431**	1		
GV	0.387**	0.306**	0.370**	0.495**	0.444**	1	
WCU	0.418**	0.324**	0.348**	0.360**	0.371**	0.406**	1

Note: ** represents a significant correlation at the 0.01 level (2-tailed).

### Direct effect tests

To examine the direct relationships among the variables, a structural equation model was constructed using JASP, and the results are presented in Table 8. When the significance level of a path coefficient was below 0.05, the direct effect between the corresponding variables was considered statistically significant, and the relevant research hypothesis was supported.

**Table 8:** SEM results.

Hypothesis	Path	Estimate (B)	Standard Error (S.E.)	z-value	p-value (Sig.)	Conclusion
H1	PFC → WCU	0.257	0.089	2.888	0.004	Supported
H2	SFC → WCU	0.006	0.072	0.089	0.929	Not supported
H3	AV → WCU	−0.003	0.089	−0.032	0.974	Not supported
H4	EV → WCU	0.035	0.069	0.508	0.611	Not supported
H5	SV → WCU	0.152	0.074	2.048	0.041	Supported
H6	GV → WCU	0.218	0.085	2.576	0.010	Supported
H7	PFC → AV	0.563	0.073	7.749	<0.001	Supported
H8	PFC → EV	0.569	0.088	6.467	<0.001	Supported
H9	SFC → SV	0.409	0.076	5.344	<0.001	Supported
H10	SFC → GV	0.368	0.063	5.832	<0.001	Supported

The SEM results showed that personal fashion consciousness was significantly and positively associated with the willingness to continue using slow fashion apparel (p = 0.004). This indicates that consumers who value individual expression and personal aesthetic preferences tend to report a stronger willingness to continue using slow fashion apparel over the long term. Thus, H1 is supported. In contrast, social fashion consciousness was not significantly associated with willingness to continue using slow fashion apparel (p = 0.929). This suggests that social evaluation or trend orientation alone was not significantly related to consumers’ willingness to continue using slow fashion apparel. Therefore, H2 is not supported.

Regarding perceived value, both social value (p = 0.041) and green value (p = 0.010) were significantly and positively associated with the willingness to continue using. This suggests that consumers’ perceptions of social recognition and environmental significance were positively related to their willingness to continue using slow fashion apparel. Thus, hypotheses H5 and H6 are supported. In contrast, aesthetic value (p = 0.974) and emotional value (p = 0.611) were not significantly associated with willingness to continue using. Therefore, H3 and H4 are not supported.

Further analysis showed that personal fashion consciousness was significantly and positively associate with aesthetic value (p < 0.001) and emotional value (p < 0.001). This suggests that consumers who prioritize individual expression and self-aesthetics tend to report higher perceptions of visual appeal and emotional resonance in slow fashion apparel, thereby supporting hypotheses H7 and H8. Simultaneously, social fashion consciousness was significantly and positively associated with social value (p < 0.001) and green value (p < 0.001). This indicates that consumers attentive to social evaluation and group norms tend to report higher perceptions of slow fashion apparel as a source of social recognition and environmental value. Thus, hypotheses H9 and H10 are both supported.

### Mediating effect tests

The results of the mediation analysis are presented in Table 9. The mediation effects were examined using a bootstrapping procedure with 5,000 resamples and 95% confidence intervals. An indirect effect was considered statistically significant when its bootstrap 95% confidence interval did not include zero. On this basis, mediation was classified as partial when the direct effect remained significant and as full when the direct effect was not significant. The results showed that the indirect effect of personal fashion consciousness on willingness to continue using through aesthetic value was not significant because its 95% confidence interval included zero. Therefore, H11 was not supported. In contrast, the indirect effect of personal fashion consciousness through emotional value was significant, while the direct effect remained statistically significant, indicating that emotional value served as a partial mediator. Thus, H12 was supported. The indirect effects of social fashion consciousness on willingness to continue using through social value and green value were both significant, and the 95% confidence intervals for both paths excluded zero. Meanwhile, the direct effect of social fashion consciousness on willingness to continue using was not significant, indicating that social value and green value both served as full mediators. Therefore, H13 and H14 were supported.

**Table 9:** Results of the mediation effect tests.

Hypothesis	Path	Indirect Effect	BootLLCI	BootULCI	Direct Effect	Total Effect	Mediation Type	Conclusion
H11	PFC → AV → WCU	0.032	−0.014	0.089	0.283***	0.350***	No mediation	Not supported
H12	PFC → EV → WCU	0.035	0.003	0.082	0.283***	0.350***	Partial mediation	Supported
H13	SFC → SV → WCU	0.027	0.002	0.067	0.082	0.140*	Full mediation	Supported
H14	SFC → GV → WCU	0.030	0.002	0.073	0.082	0.140*	Full mediation	Supported

**Note:** *** indicates significance at the 0.001 level; * indicates significance at the 0.05 level.

## Conclusions and Discussion

Against the backdrop of sustainable consumption, this study developed a conceptual model examining the relationships among fashion consciousness, perceived value, and willingness to continue using slow fashion apparel, and empirically tested the model using data from Gen Z consumers. The findings indicate that: 1) personal fashion consciousness was significantly and positively associated with willingness to continue using slow fashion apparel, whereas the direct relationship between social fashion consciousness and willingness to continue using was not significant; 2) among the perceived value dimensions, social value and green value were significantly and positively associated with willingness to continue using slow fashion apparel, whereas the direct relationships of aesthetic value and emotional value with willingness to continue using were not significant; 3) personal fashion consciousness was significantly and positively associated with both aesthetic value and emotional value, while social fashion consciousness was significantly and positively associated with both social value and green value; and 4) the mediation analysis further showed that emotional value partially mediated the relationship between personal fashion consciousness and willingness to continue using, whereas social value and green value fully mediated the relationship between social fashion consciousness and willingness to continue using. The mediating role of aesthetic value was not supported.

No significant direct relationship was observed between social fashion consciousness and willingness to continue using, indicating that consumers’ attention to fashion trends and social evaluations was not directly associated with long-term wearing behavior. Compared with purchase decisions, continued use is a post-purchase behavior that places greater emphasis on repeated wear, long-term retention, and active maintenance. This study further found that social fashion consciousness was significantly associated with willingness to continue using through social value and green value. This suggests that social fashion consciousness is more likely to be associated with willingness to continue using when it is accompanied by perceptions of social recognition and environmental significance.

In addition, aesthetic value was neither significantly associated with willingness to continue using nor a significant mediator between personal fashion consciousness and willingness to continue using, indicating that visual appeal alone may be insufficient to encourage long-term use. Whether slow fashion apparel is continuously used may also depend on factors such as durability, comfort, and styling versatility. Although emotional value was not directly associated with willingness to continue using, it partially mediated the relationship between personal fashion consciousness and willingness to continue using. This finding suggests that value experiences arising from individual expression, self-identity, and emotional connection may strengthen the association between personal fashion consciousness and willingness to continue using.

### Theoretical implications

This study has the following theoretical implications. First, it applies the SOR theory to the context of continued use of slow fashion apparel and develops a theoretical framework linking fashion consciousness, perceived value, and willingness to continue using. By shifting the research focus from purchase intention and purchasing behavior to post-purchase continued use, this study broadens the application of the SOR theory in the context of sustainable consumption research and provides a new theoretical perspective for explaining consumers’ continued use of slow fashion apparel. Second, this study advances the multidimensional understanding of perceived value. The findings reveal that different value dimensions play distinct roles during the continued-use stage. This result suggests that aesthetic and emotional experiences may not necessarily be sufficient to explain long-term use behavior and further suggests that value attributes that are attractive at the purchase stage may not independently explain consumers’ post-purchase willingness to continue using apparel.

### Practical implications

Based on the findings, practical recommendations are proposed from four perspectives: product design, marketing communication, service systems, and policy support.

1) Develop products that balance individual expression with long-term usability. Because personal fashion consciousness was significantly and positively associated with willingness to continue using slow fashion apparel, brands should reduce their reliance on short-lived fashion trends during product development and place greater emphasis on timeless silhouettes, styling versatility, cross-seasonal wearability, and a moderate degree of design distinctiveness. Products should not only express individual style but also accommodate long-term use across different occasions. Brands may also provide personalized styling advice, partial customization, and replaceable accessories to help consumers maintain a sense of novelty without frequently replacing entire garments.
2) Improve the transparency and credibility of green information. Brands and marketers should avoid relying solely on broad claims such as “eco-friendly” or “sustainable.” Instead, they should provide specific information about material sources, production processes, durability, care methods, and product life cycles. Product labels, QR codes, official websites, and digital product records can be used to present raw-material certifications, estimated product lifespans, repair options, and environmental information, enabling consumers to identify the green attributes of slow fashion apparel more clearly. Communication strategies should also emphasize long-term wear, repair and maintenance, and circular use to support more informed and responsible consumption decisions.
3) Enhance the visibility of social value through community interaction. Brands and marketers can establish sustainable styling communities, organize long-term campaigns such as “one garment, multiple outfits,” and encourage consumers to share their experiences of repeated wear, garment alteration, repair, and styling. Compared with simply presenting new products, authentic consumer stories, records of wearing frequency, and long-term use cases may better communicate the image of slow fashion as a lifestyle associated with taste, responsibility, and social recognition.
4) Improve services that support long-term use and circular consumption. Brands can support continued use through repair, maintenance, garment take-back, rental, and trade-in services, while retaining commonly used replacement parts over the long term. Standardized repair options at reasonable prices should be offered for areas that are particularly vulnerable to wear. For classic products, brands should continue to stock matching fabrics, buttons, zippers, and other components. Policymakers can encourage enterprises to improve product information transparency and after-sales service capabilities through green product standards, durability and repairability labels, circular textile certification, and support for repair services. Public education and industry guidelines should also promote responsible purchasing, extended product use, and standardized recycling, which may contribute to a more supportive institutional and social environment for the long-term use of slow fashion apparel.

### Limitations and future research

Although this study examined the factors associated with of Generation Z consumers’ willingness to continue using slow fashion apparel, it still has several limitations. 1) This study used cross-sectional survey data. The results mainly reflect respondents’ psychological perceptions and behavioral intentions at a specific point in time. Therefore, it is difficult to reveal the dynamic changes in the study variables. Future research could conduct longitudinal tracking studies further examine changes in consumers’ value perceptions and behaviors across the stages of purchase, use, and disposal. 2) This study mainly examined the effects of fashion consciousness and perceived value on willingness to continue using. However, willingness to continue using slow fashion apparel may also be associated with other factors, such as price sensitivity, product durability, brand trust, consumption habits, environmental knowledge, and actual usage contexts. Future research could include these variables to build a more comprehensive model of willingness to continue using slow fashion apparel. 3) This study measured willingness to continue using rather than actual continued use behavior. There may still be a gap between intention and actual behavior. Future research could include objective indicators, such as actual wearing frequency, clothing retention time, repair behavior, second-hand resale behavior, or used-clothing recycling behavior. This would improve the explanatory power of the research findings.

## Supporting information

S1 File Raw images. (PNG)

S1 Data Anonymized survey dataset underlying the findings of this study. This file contains the anonymized raw questionnaire responses used for all statistical analyses.
(XLSX)
